# Functions of Thrombospondin-1 in the Tumor Microenvironment

**DOI:** 10.3390/ijms22094570

**Published:** 2021-04-27

**Authors:** Sukhbir Kaur, Steven M. Bronson, Dipasmita Pal-Nath, Thomas W. Miller, David R. Soto-Pantoja, David D. Roberts

**Affiliations:** 1 Laboratory of Pathology, Center for Cancer Research, National Cancer Institute, NIH, Bethesda, MD 20892, USA; Sukhbir.Kaur@nih.gov (S.K.); dipasmita.palnath@nih.gov (D.P.-N.); 2 Department of Internal Medicine, Section of Molecular Medicine, Comprehensive Cancer Center, Wake Forest School of Medicine, Winston-Salem, NC 27101, USA; sbronson@wakehealth.edu; 3 Centre de Recherche en Cancérologie de Marseille, Institut Paoli-Calmettes, 13273 Marseille, France; 4 Department of Surgery and Department of Cancer Biology, Comprehensive Cancer Center, Wake Forest School of Medicine, Winston-Salem, NC 27101, USA

**Keywords:** angiogenesis, cytotoxic T cells, natural killer cells, CD36, CD47, integrins, tumor-initiating cells, autophagy, nitric oxide, transforming growth factor-β1

## Abstract

The identification of thrombospondin-1 as an angiogenesis inhibitor in 1990 prompted interest in its role in cancer biology and potential as a therapeutic target. Decreased thrombospondin-1 mRNA and protein expression are associated with progression in several cancers, while expression by nonmalignant cells in the tumor microenvironment and circulating levels in cancer patients can be elevated. *THBS1* is not a tumor suppressor gene, but the regulation of its expression in malignant cells by oncogenes and tumor suppressor genes mediates some of their effects on carcinogenesis, tumor progression, and metastasis. In addition to regulating angiogenesis and perfusion of the tumor vasculature, thrombospondin-1 limits antitumor immunity by CD47-dependent regulation of innate and adaptive immune cells. Conversely, thrombospondin-1 is a component of particles released by immune cells that mediate tumor cell killing. Thrombospondin-1 differentially regulates the sensitivity of malignant and nonmalignant cells to genotoxic stress caused by radiotherapy and chemotherapy. The diverse activities of thrombospondin-1 to regulate autophagy, senescence, stem cell maintenance, extracellular vesicle function, and metabolic responses to ischemic and genotoxic stress are mediated by several cell surface receptors and by regulating the functions of several secreted proteins. This review highlights progress in understanding thrombospondin-1 functions in cancer and the challenges that remain in harnessing its therapeutic potential.

## 1. Introduction

Cancer has been described as a wound that does not heal [1]. Despite many similarities between the wound and tumor microenvironments, exploitable differences in the recruited cell types and their secreted extracellular matrix products have been identified as contributors to cancer progression and potential therapeutic targets. Our interest in studying the role of the secreted protein thrombospondin-1 (TSP1, encoded by *THBS1*) in the tumor microenvironment arose from our observation that TSP1 expression decreased during malignant progression in melanoma and breast carcinoma cell lines. The expression of oncogenic forms of K-Ras, H-Ras, and N-Ras in bronchial epithelial cells also suppressed TSP1 mRNA and protein expression [2]. Conversely, re-expression of TSP1 impaired tumor growth and metastasis in several types of cancer, including melanoma, glioblastoma, prostate carcinoma, squamous cell carcinoma, and cervical carcinoma [3,4,5,6,7]. Other oncogenes were subsequently found to negatively regulate TSP1 expression, including *MYC* [8,9]. Conversely, inactivation of tumor suppressor genes such as *TP53* and *RB1* suppressed TSP1 expression [10,11,12,13,14,15]. Deletion of *THBS1* is a rare event in most human cancers, and the observed loss of expression largely results from epigenetic effects of the altered oncogenes and tumor suppressor genes [16]. Despite the general loss of TSP1 expression in malignant cells, elevated circulating levels of TSP1 in blood have been reported in several human and murine cancers [10]. TSP1 expression is also induced in the wound microenvironment [17]. The relevance of TSP1 in the wound/cancer dichotomy was further suggested by a report that showed TSP1 mRNA is upregulated in renal tissue regeneration but downregulated in renal cell carcinoma [18].

In addition to an intrinsic role for TSP1 expressed by tumor cells, increased growth of B16 melanomas and F9 testicular teratocarcinomas was observed when implanted in syngeneic mouse strains lacking *Thbs1* [19]. As TSP1 is a secreted protein, its abundance in the tumor microenvironment depends on both tumor and stromal cell expression. TSP1 in the tumor microenvironment can influence the behavior of multiple cell types that regulate tumor growth and metastasis. In addition to being regulated by oncogenes and tumor suppressor genes, TSP1 in the microenvironment can mediate feedback regulation of their expression, as demonstrated for p53 and Myc [20]

**Figure 1:**
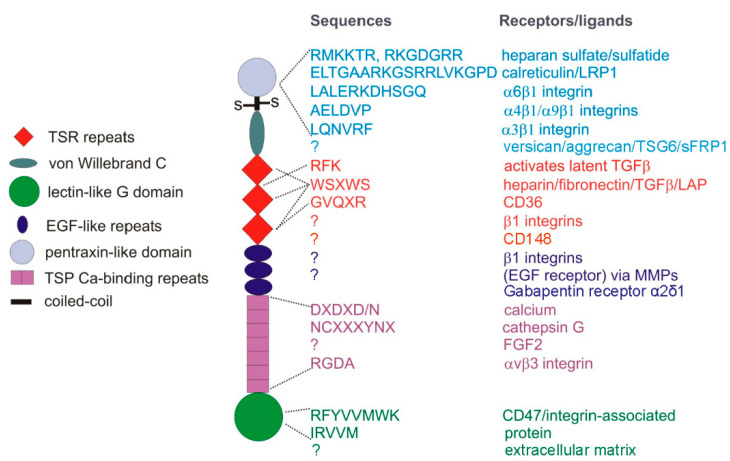
TSP1 subunit domains and their cell surface receptors or extracellular ligands. TSP1 is a ~450 kDa homotrimer of subunits linked by disulfide bonds near the N-terminal pentraxin-like domain. Type 1 TSP1 repeats (TSR), EGF-like, and calcium-binding repeats form the central stalk region of TSP1, connecting the N- and C-terminal globular domains.

Consistent with the complexity of function for other matricellular proteins, both protective and tumor-promoting functions of TSP1 have also been reported. Divergent roles of TSP1 can be mediated by engaging different TSP1 receptors (Figure 1). In cases where cells express multiple TSP1 receptors, responses to TSP1 can be biphasic. For example, by engaging several integrins, TSP1 can promote endothelial cell motility and proliferative responses, whereas engaging CD47 on the same cells inhibits the same responses [10,21,22].

This review focuses on the direct effects of TSP1 on tumor cells, the vascular cells that enable the delivery of oxygen and nutrients required for tumor growth, and host immune cells that can mediate effective antitumor immunity but also produce factors that protect some tumors from immune clearance and facilitate metastatic spread.

## 2. Functions of TSP1 Receptors and Secreted Interaction Partners

Understanding the divergent functions of TSP1 in cancer requires an appreciation of the multiple TSP1 receptors that are expressed on each cell type in the tumor microenvironment as well as the secreted factors that interact with TSP1 and mediate some of its functions (Figure 1). Domains of TSP1 and specific peptide sequences have been identified that are recognized by some of its receptors. As TSP1 is a substrate for several proteases in the tumor microenvironment, fragments of TSP1 that engage specific subsets of receptors may have biologic activities that differ from those of intact TSP1 [23,24].

### 2.1. Cell Surface Receptors

#### 2.1.1. Integrins

Several integrins have been identified that recognize TSP1 (Table 1), and binding sites in TSP1 recognized by several of these have been identified (Figure 1). These mediate the promotion of adhesion and migration of tumor cells, vascular cells, and T cells by TSP1 [25,26,27]. Several β1 integrins mediate the pro-angiogenic activities of N-terminal fragments of TSP1 [23,24,28,29]. The RGD sequence in the calcium-binding repeats of TSP1 is recognized by αvβ3, but its accessibility is regulated by disulfide bond isomerization in this domain [30].

#### 2.1.2. CD36

Binding sites for CD36 were identified in the central type 1 repeats of TSP1 [35]. CD36 is abundant on macrophages and selectively expressed on microvascular endothelial cells, where it mediates an anti-angiogenic activity of TSP1 [35]. Peptide mimetics based on one of these sites demonstrated anti-angiogenic and anti-tumor activities in preclinical studies [43,44], but not in human efficacy trials [45,46,47].

#### 2.1.3. CD47

Two CD47-binding sequences were identified in the C-terminal domain of TSP1 [48]. However, a recombinant region of TSP1 containing these sequences did not bind to a nonglycosylated extracellular domain of CD47 based on surface plasmon resonance [49]. Subsequent studies confirmed that high-affinity binding of the C-terminal domain of TSP1 to cells requires CD47 and demonstrated that glycosaminoglycan modification of CD47 is necessary for TSP1 signaling [50,51]. A recent super-resolution microscopy study indicated that high-affinity binding of TSP1 is mediated by clustering of CD47 on aged red blood cells [52]. Expression of CD47 is broadly elevated in cancers, and in some cancers, higher CD47 expression is associated with poor survival [53]. Expression of CD47 in the tumor microenvironment is regulated by oncogenes, including *MYC* [15]. CD47-blocking antibodies have shown efficacy in preclinical cancer models and early clinical trials, but these act primarily by blocking CD47 interactions with SIRPα rather than TSP1 [54].

#### 2.1.4. Calreticulin/LRP1

The scavenger receptor low density lipoprotein receptor-related protein-1 (LRP1) mediates uptake of TSP1 by several cell types that result in its degradation [55]. The interaction between TSP1 and LRP1 is mediated by calreticulin, which signals disassembly of focal adhesions in some cell types [56]. Several functions of LRP1 as a TSP1 receptor have been identified relevant to the tumor microenvironment. Interaction of a TSP1 fragment produced by T cells with calreticulin/LRP1 regulated their motility, integrin-dependent adhesion, and interaction with antigen-presenting cells [40,57]. A complex containing LRP1, TSP1, and annexin A6 was recently identified as being produced by cancer-associated fibroblasts in pancreatic ductal adenocarcinoma [41]. This complex was associated with extracellular vesicles (EVs) released by the fibroblasts taken up by the tumor cells. Circulating EVs bearing annexin A6 were identified as a biomarker for pancreatic carcinoma, and inhibition of their function in a mouse model decreased metastasis.

### 2.2. Functional Interactions of TSP1 with Other Secreted Proteins

#### 2.2.1. TGFβ

TSP1 is one of several factors that can mediate the activation of latent-TGFβ1 by dissociating the latency-associated peptide [58]. In the context of cancer, TGFβ stimulates the growth of some cancers and suppresses antitumor immunity [59,60]. Inhibition of TSP1-mediated activation of latent TGFβ was effective in a preclinical model to treat multiple myeloma [61].

#### 2.2.2. Proteases

In addition to being a substrate for several extracellular proteases, including thrombin, TSP1 was identified as a direct inhibitor of several proteases, including plasmin, neutrophil elastase, tissue factor pathway inhibitor, and cathepsin G [62,63]. TSP1 is also a competitive inhibitor of von Willebrand factor’s cleavage by ADAMTS13 [64]. Conversely, TSP1 can increase the activity of matrix metalloproteinases by limiting the production of tissue inhibitors of metalloproteinases-1 (TIMP1) [65] and by β1 integrin-dependent induction of MMP2, MMP7, and MMP9 expression in T cells [25].

#### 2.2.3. Angiogenic Growth Factors

Direct binding of TSP1 to fibroblast growth factor-2 and vascular endothelial growth factor-A (VEGF) contributes to its anti-angiogenic activities under certain conditions [66,67]. Interaction with platelet-derived growth factor (PDGF) modulates vascular smooth muscle recruitment during angiogenesis [68,69]. Interaction with PDGF also enhances mesenchymal stromal cell formation [70].

#### 2.2.4. sFRP1

In addition to containing heparin and integrin binding sites, the N-terminal pentraxin-like module of TSP1 interacts specifically with several members of the hyaladherin family that contain link modules and with secreted frizzled-related protein (sFRP)-1 [71,72] (Figure 1). sFRP1 inhibited breast carcinoma cell adhesion on immobilized TSP1 mediated by α3β1 integrin and inhibited migration of the same cells induced by TSP1 [72].

## 3. TSP1 Regulation of Angiogenesis and Tumor Perfusion

### 3.1. Inhibition and Stimulation of Angiogenesis

Early efforts to define the underlying mechanism by which the loss of TSP1 in the tumor microenvironment contributes to cancer progression resulted in three independent reports in 1990 identifying TSP1 as an angiogenesis inhibitor [21,73,74]. TSP1 is a potent inhibitor of endothelial cell migration and proliferation and an inducer of endothelial apoptosis [21,75]. Consistent with these results, TSP1 inhibited angiogenesis in rat cornea [74], chicken chorioallantoic membrane [76], and muscle explants in 3D culture [77]. Evidence from mouse models demonstrated that elevating TSP1 expression inhibits tumor growth with a corresponding decrease in vascular density [3,11,78].

CD36 was the first TSP1 receptor identified to mediate its anti-angiogenic activity [35], and peptide mimetic drugs derived from a sequence in the second TSR repeat of TSP1 that binds to CD36 (Figure 1) have been developed to inhibit tumor angiogenesis [43]. However, some observations were not consistent with the hypothesis that TSP1 functions solely as an angiogenesis inhibitor. If true, one would expect TSP1 to be downregulated during wound repair as well as in cancer. One would also predict that the loss of this inhibitor would accelerate wound repair in a *Thbs1^−/−^* mouse. Instead, TSP1 was rapidly up-regulated following excisional skin wounding [79], and the *Thbs1^−/−^* mouse exhibited delayed wound closure in this model [80]. Furthermore, TSP1 antisense oligonucleotides delayed repair of excisional skin wounds in wild-type (WT) mice [79]. This activity of TSP1 contrasts with TSP2, a closely related protein that also inhibits angiogenesis and engages CD36 [81,82,83]. TSP2 is expressed only late in wound closure, and *Thbs2^−/−^* mice showed the predicted accelerated excisional wound repair [80]. Agah et al. rationalized the unexpected wound repair phenotype of the *Thbs1^−/−^* mouse by invoking another TSP1 responsive cell type. They found that macrophage infiltration was impaired in the absence of TSP1, and chemokines’ levels essential for wound repair such as MCP1 were lower. Therefore, they proposed that the dominant activity of TSP1 induced early in excisional wound repair is not to limit angiogenesis but instead to recruit monocytes into the wound.

**Figure 2:**
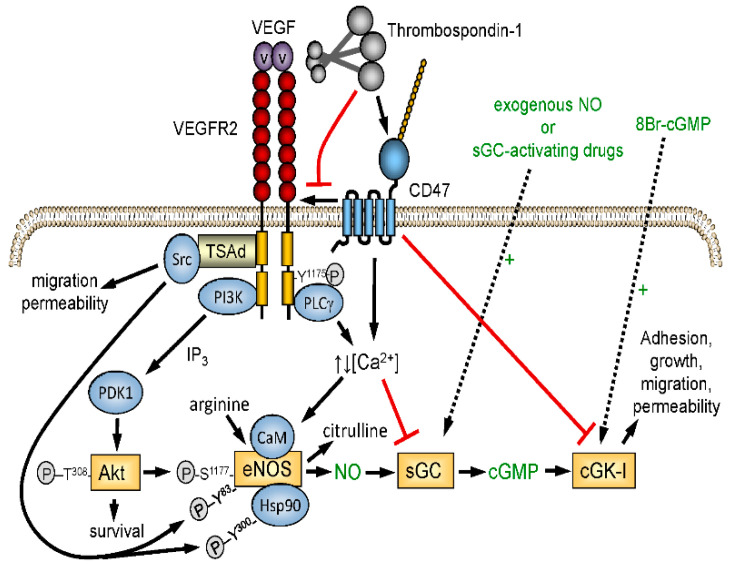
Redundant regulation of NO signaling by TSP1–CD47 interaction. Endogenous NO synthesis is stimulated via Akt-mediated phosphorylation of endothelial nitric oxide synthase (eNOS) downstream of the VEGF receptor VEGFR2. Ligation of CD47 by TSP1 also inhibits activation of soluble guanylyl cyclase (sGC) mediated by endogenous or exogenous NO. TSP1 also inhibits calcium-dependent activation of eNOS and NO signaling downstream of cGMP by inhibiting cGMP-dependent protein kinase (cGK-1).

The excisional skin wound model lacked ischemic stress, which reveals a more critical function of TSP1 in limiting the angiogenic response required for wound repair. Nitric oxide (NO)/cGMP signaling is a central regulator of angiogenesis and tissue perfusion under ischemia, and TSP1 signaling through its receptor CD47 potently inhibits NO biosynthesis and signaling in vascular cells [37] (Figure 2). *Thbs1^−/−^* and *cd47^−/−^* mice exhibit enhanced vascular responses to NO and improved recovery from ischemic injuries [84]. Blocking CD47 expression or function in WT mice, rats, and miniature pigs improved recovery from ischemic injuries [85,86,87,88,89,90]. TSP1 inhibition of the angiogenic response to NO donors was lost in muscle explants from *cd47^−/−^* mice but preserved in *cd36^−/−^* mice [91]. We established physiological and pathophysiological roles for CD47 in mediating TSP1 signaling in endothelial cells, vascular smooth muscle cells, and platelets [77,84,92,93]. Therefore, the elevated TSP1 in wounds has two acute activities in addition to its long-term regulation of angiogenesis and vascular remodeling: (1) TSP1 is a potent vasoconstrictor that acutely limits bleeding but can be counterproductive for tissue survival under ischemic stress, and (2) TSP1 is an autocrine factor released by platelets that promotes hemostasis.

These studies provide additional insights into why TSP1 is downregulated in cancers. As TSP1 limits angiogenesis and perfusion, its local expression is clearly a disadvantage to a growing tumor [10]. Loss of TSP1 expression may also decrease the thrombogenic potential of the tumor vasculature by maximizing the anti-thrombotic activity of NO produced by the tumor and its stroma.

### 3.2. Vascular Perfusion of Tumors and the Steal Effect

CD47-dependent antagonism of NO signaling by TSP1 limits the perfusion of healthy tissues, but this signaling is impaired in the tumor vasculature [94]. In the closed system of vascular physiology, vasodilation in one vascular bed can “steal” blood flow from another vascular bed fed by the same perfusing artery [95]. Conversely, TSP1-mediated constriction of healthy tissue vasculature can result in increased perfusion of tumors, providing selective pressure for the elevated circulating TSP1 levels observed in some cancers [10].

### 3.3. Endothelial Cell Apoptosis

TSP1 and its type 1 repeats induce apoptosis of endothelial cells [75]. CD36 and CD47 have been implicated in mediating TSP1 apoptotic signaling [96,97]. The induction of TNFα expression by TSP1 in endothelial cells may mediate cell death based on a requirement for tumor necrosis factor receptor-1 [98]. In the tumor microenvironment, TSP1-induced endothelial cell apoptosis contributes to the antitumor activity of low-dose cyclophosphamide treatment [99].

## 4. TSP1 and Antitumor Immunity

### 4.1. Regulation of T Cell Immunity

Our interest in the regulation of anti-tumor immunity by TSP1 began with the observation that TSP1 globally suppresses changes in gene expression that are induced by T cell antigen receptor (TCR) signaling [100], which we subsequently found to require CD47 [25,51,101]. Others reported that engaging CD47 limits T cell-dependent inflammation in vivo [102], induces T cell apoptosis in the context of TCR signaling [103], and induces CD4^+^ T cell differentiation into regulatory T cells [104]. The latter study implied that TSP1 has the same activity, but the only evidence presented involved a TSP1 peptide analog with limited specificity [37,105].

Immune cell responses to TSP1 are defined by integrating signals from several cell surface receptors [25,106,107,108]. Jurkat T cells have been a valuable model for defining such cross-talk because somatic mutants are available that lack the TSP1 receptors α4β1 integrin, CD47, and several signaling molecules downstream of these receptors [25,107]. These tools and T cells isolated from transgenic mice lacking TSP1 or CD47 enabled us to confirm or exclude the contribution of each receptor to specific T cell responses in vitro and immune responses in vivo [25,51,101,107,109,110].

TSP1-mediated CD47 signaling limits antigen-dependent T cell activation by several mechanisms. By inhibiting signal transduction downstream of the T cell receptor, TSP1 inhibits the expression of genes encoding IL-2 and TNFα that can stimulate proliferation and activation of other immune cells and the α-subunit of the IL-2 receptor (CD25), which limits the ability of T cells exposed to TSP1 to respond to exogenous IL-2. TSP1 also limits the expression of cystathionine β-synthase, which is induced during T cell activation and produces the diffusible mediator H_2_S that acts on the T cell cytoskeleton to regulate T cell polarization required for immunological synapse formation [101,111].

Sensitivity of B16 melanomas in immune-competent mice to ablation by tumor irradiation was enhanced when either *Thbs1* or *Cd47* were disrupted in the tumor microenvironment [112,113]. Subsequent studies revealed that this increased sensitivity to ionizing radiation requires CD8^+^ T cells. Furthermore, therapeutic blockade of TSP1/CD47 signaling using antibodies or antisense CD47 knockdown enhances antigen-dependent killing of irradiated tumor cells by mouse and human CD8^+^ T cells in vitro and tumors in athymic mice following adoptive transfer of tumor-specific CD8^+^ T cells [113,114]. Therefore, CD47 on CD8^+^ T cells functions as an adaptive immune checkpoint that mediates TSP1-dependent inhibition of tumor cell killing.

### 4.2. TSP1 Regulation of Innate Immune Cells

TSP1 also regulates innate immune cells relevant to the tumor microenvironment. Early studies examined TSP1 effects on neutrophil oxidative burst and found that TSP1 synergizes with a formylated bacterial peptide to stimulate an oxidative burst response [115,116]. Others identified inhibitory TSP1 functions in NK cells [117,118], dendritic cells [119,120,121,122,123], and monocytes [108,117,124,125,126,127]. Functions of TSP1 in other aspects of immune responses include modulation of TLR3-mediated inflammatory signaling in VSMC [71].

Several studies have reported the regulation of myeloid cell functions by TSP1 in the tumor microenvironment. Secretion of TSP1 by tumor cells increased macrophage recruitment and increased M1 polarization in a melanoma xenograft model [32]. TSP1 increased oxidative killing of tumor cells by macrophages in vitro. Tumors with low metastatic potential were reported to induce TSP1 expression by bone marrow-derived Gr1**^+^** myeloid cells, and targeted deletion of *Thbs1* in myeloid cells abolished their anti-metastatic activity [128]. Myeloid-derived suppressor cells released EVs that contained TSP1, and a TSP1 antibody specifically inhibited migration of myeloid-derived suppressor cells induced by these EVs [129]. Finally, a protective role of TSP1 to limit UVB-induced skin carcinogenesis was attributed to an anti-inflammatory function that limited the expansion of myeloid progenitor cells in the neutrophil lineage [130].

**Figure 3:**
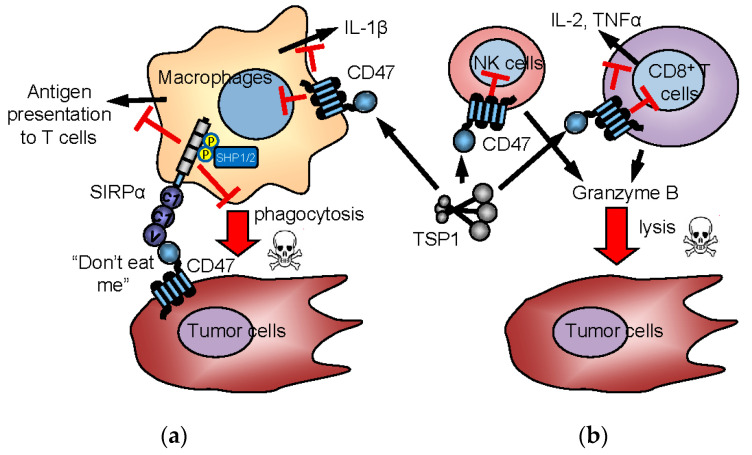
Innate and adaptive immune checkpoint functions of CD47 and TSP1. In the “don’t eat me” model (**a**), CD47 on tumor cells interacts with SIRPα on macrophages to induce inhibitory signaling, preventing the phagocytic killing of tumor cells. CD47 is also expressed by immune cells and mediates TSP1 signaling in immune cells. TSP1 interaction with CD47 on NK cells and cytotoxic T cells inhibits their activation and limits granzyme B production that mediates antigen-dependent lysis of tumor cells (**b**). Inhibitory TSP1 signaling mediated by CD47 on macrophages and dendritic cells limits their presentation of antigens to T cells and limits macrophage production of IL-1β.

Interest in CD47 as a regulator of anti-tumor innate immunity was heightened by reports that a CD47 blocking antibody enhances NK- and macrophage-mediated killing of tumor cells [131,132]. CD47 was originally discovered as a tumor-associated antigen for ovarian cancer [133], but its pathophysiological significance was unclear. Elevated CD47 expression on tumor cells is now recognized as a general mechanism to evade host innate immunity for various solid tumors and hematologic malignancies [131,132,134,135,136,137,138,139,140,141]. The prevailing model proposes that high CD47 expression on tumor cells engages the inhibitory counter-receptor SIRPα on phagocytes to prevent tumor cell killing (Figure 3a). CD47-induced SIRPα signaling in macrophages and dendritic cells indirectly enhances anti-tumor adaptive T cell immune responses [142]. However, CD47 blockade can also enhance antibody-dependent cellular cytotoxicity independent of SIRPα signaling [143]. Enhanced macrophage clearance of tumor cells damaged by irradiation was a plausible mechanism to account for the synergism we observed between radiation and CD47 knockdown in syngeneic tumor models [144]. However, further studies revealed that CD47 signaling in murine and human CD8^+^ T cells directly inhibits their antigen-dependent killing of tumor cells (Figure 3b) [113,114].

Conversely, in the absence of CD8^+^ T cells, blocking CD47 does not inhibit tumor growth in syngeneic models [113,145,146], and genetic disruption of SIRPα signaling in macrophages did not impair syngeneic tumor growth [143]. Thus, complete loss of the “don’t eat me” signal is insufficient to prevent tumor growth in the absence of cytotoxic T cell activity or secondary stimuli to induce innate immune clearance [147]. The tumor cell response to damage induced by ionizing radiation provides such a signal to enhance CD47-induced tumor clearance in immune-competent mice. This clearance also requires cytotoxic T cell activity [113].

Several humanized CD47 antibodies and SIRPα decoys have entered clinical trials as cancer therapeutics [54,148,149,150,151]. All clinical CD47 antibodies block SIRPα binding to CD47 and are intended to enhance innate immune-mediated clearance of tumors [138,140,152,153,154]. Likewise, anti-SIRPα antibodies have shown preclinical activity as a single agent or in combination with a tumor-targeted agent [54]. However, with respect to CD47-directed agents, several findings raise doubts about an exclusive role of SIRPα and macrophages in the antitumor activity of CD47 blockade. We and others have reported that CD47 antibodies exhibit anti-tumor activities independent of SIRPα signaling [113,143,147,155,156,157,158].

### 4.3. CD47 and TSP1 Signaling in Macrophages

Recent studies have emphasized the role of the inhibitory receptor SIRPα in regulating macrophage phagocytosis of tumor cells that highly express CD47 [141]. Still, macrophages also express CD47, and physiological concentrations of TSP1 limit the induction by lipopolysaccharide (LPS) of IL-1β mRNA and total IL-1β protein production by human macrophages [159]. This inhibition could be explained by the ability of TSP1 binding to disrupt the interaction between CD47 and CD14, thereby limiting activation of NFκB/AP-1 by LPS. Only the CD47-binding domain of TSP1 exhibited this activity. In contrast, CD47, CD36, and integrin-binding domains of TSP1 independently enhanced the inflammasome-dependent maturation of IL-1β in human THP-1 monocyte-derived macrophages. Correspondingly, mouse bone marrow-derived macrophages lacking either TSP1 or CD47 exhibited diminished induction of mature IL-1β in response to LPS. Loss of CD47 also limited LPS induction of IL-1β, NLRP3, and caspase-1 mRNAs. These data demonstrate that TSP1 exerts CD47-dependent and -independent pro-and anti-inflammatory effects on the IL-1β pathway in macrophages.

### 4.4. Intrinsic Functions of CD47 in NK Cells

TSP1 and CD47 also have cell-intrinsic roles in regulating NK cells. TSP1 inhibited early NK cell proliferation and enhanced late expansion, but a role for CD47 was not examined [117]. CD47 as a SIRPα counter-receptor enabled the engraftment of NK precursors in mice reconstituted with a human immune system [160]. Treatment with an inhibitory CD47 antibody increased NK cell killing of human head-and-neck squamous carcinoma cells in vitro [131]. However, NK cells were not known to express SIRPα, and the mechanism was unclear at the time. SIRPα expression was recently shown to be induced in activated NK cells, and ligation by CD47 alters NK cell function [161]. Depletion of NK cells similarly attenuated the anti-tumor activity of a SIRPα blocking antibody in a syngeneic murine renal carcinoma model, but the same antibody did not inhibit NK killing of the tumor cells in vitro, further supporting a SIRPα-independent function of CD47 in NK cells [162]. We found an increased abundance of lineage-negative cells within the spleen of *Cd47^−/−^* mice and discovered these to be immature NK cells [163]. This led us to further investigate the role of CD47 in NK cell homeostasis.

*Cd47^−/−^* mice exhibited depletion of NK precursors in bone marrow, consistent with the antiphagocytic function of CD47. In contrast, antisense CD47 knockdown or gene disruption resulted in a dose-dependent accumulation of immature and mature NK cells in spleen. Mature *cd47^−/−^* NK cells exhibited increased expression of NK effector and interferon gene signatures and an increased proliferative response to interleukin-15 in vitro. *Cd47^−/−^* mice showed no defect in their early response to acute Armstrong lymphocytic choriomeningitis virus (LCMV) infection but were moderately impaired in controlling chronic Clone-13 LCMV infection. This was associated with depletion of splenic NK cells and loss of effector cytokine and interferon response gene expression in *cd47^−/−^* NK cells. Broad CD47-dependent differences in NK activation, survival, and exhaustion pathways were observed in NK cell transcriptional signatures in LCMV infected mice. These data identify CD47 as a cell-intrinsic and systemic regulator of NK cell homeostasis and NK cell function in responding to a viral infection. Consistent with our data, a recent study found increased NK cell activation following induction of atherosclerosis in *cd47^−/−^* mice [118].

Extending these findings to the role of CD47 in cancer, we examined the NK cells in syngeneic B16 melanomas growing in WT versus *cd47^−/−^* mice [164]. Elevated CD47 expression in some cancers is associated with decreased survival and limited clearance by phagocytes expressing the CD47 counterreceptor SIRPα [140,141]. In contrast, we found that elevated CD47 mRNA expression in human melanomas is associated with improved survival [164]. Gene-expression data identified a potential mechanism for this apparent protective function and suggested that high CD47 expression increases NK cell recruitment into the tumor microenvironment. The CD47 ligand TSP1 inhibited NK cell proliferation in vitro and the induction of CD69 expression [164]. *Cd47^−/−^* NK cells correspondingly displayed augmented effector phenotypes, indicating an inhibitory function of CD47 on NK cells. Treating human NK cells with a CD47 antibody that blocks TSP1 binding abrogated its inhibitory effect on NK cell proliferation [164]. Similarly, treating wild-type mice with a CD47 antibody that blocks TSP1 binding delayed B16 melanoma growth, associating with increased NK cell recruitment and increased granzyme B and interferon-γ levels in intratumoral NK but not CD8^+^ T cells [164].

However, B16 melanomas grew faster in *cd47^−/−^* than in WT mice [164]. Melanoma-bearing *cd47^−/−^* mice exhibited decreased splenic NK cell numbers, with impaired effector protein expression and elevated exhaustion markers. Proapoptotic gene expression in *cd47^−/−^* NK cells was associated with stress-mediated increases in mitochondrial proton leak, reactive oxygen species, and apoptosis [164]. Global gene-expression profiling in NK cells from tumor-bearing mice identified CD47-dependent transcriptional responses that regulate systemic NK activation and exhaustion. Therefore, CD47 positively and negatively regulates NK cell function, and therapeutic antibodies that block inhibitory TSP1-CD47 signaling can enhance NK immune surveillance of melanomas.

### 4.5. TSP1 in Supramolecular Attack Particles

A novel role for TSP1 was recently identified in the supramolecular attack particles released by cytotoxic T cells and NK cells that kill target tumor cells [165,166]. A fragment of TSP1 was identified as a component of the outer shell of these particles that deliver perforin and granzyme-B from their core to kill target cells. Notably, CRISPR-mediated deletion of TSP1 impaired the cytotoxic activity of CD8^+^ T cells, indicating that TSP1 plays an intrinsic role in the cytotoxic function of supramolecular attack particles.

## 5. Direct Effects of TSP1 on Tumor Cells

### 5.1. Cell Adhesion and Migration

The integrin α3β1 mediates positive effects of TSP1 on the adhesion and migration of breast carcinoma cells [167]. TSP1 also stimulates chemotaxis of melanoma, squamous carcinoma, and promyelocytic leukemia cells [168,169,170,171].

CD47 mediates positive effects of TSP1 on proliferation, survival, and migration of cutaneous T-cell lymphoma cells [172]. These effects involved increased phosphorylation of ERK1/2 and AKT and expression of survivin. Similarly, TSP1 increased the invasive behavior of follicular thyroid carcinoma cells by increasing urokinase-dependent proteolytic activity [173]. In papillary thyroid carcinoma, the prevalent B-Raf(V600E) mutation promoted the invasive behavior in part by inducing expression of TSP1 [174].

Loss of the von Hippel-Lindau tumor suppressor gene (*VHL*) is a significant driver of renal cell carcinoma, in part by stabilizing hypoxia-inducible factors (HIF). However, loss or mutation of *VHL* was recently shown to decrease TSP1 expression independent of HIF [175]. The *VHL*-dependent loss of TSP1 expression in renal clear cell carcinoma increased the migratory behavior of these cells. Thus, TSP1 in the tumor microenvironment may regulate tumor invasion through direct and indirect pathways.

### 5.2. Tumor Cell Death/Senescence

Treatment with TSP1 induces death of several types of cancer cells. The C-terminal domain of TSP1 induced caspase-independent death of promyelocytic leukemia cells [176]. TSP1 induced CD47-dependent death of T lymphoma and breast carcinoma cells [103,156]. Peptide mimetics of TSP1 and CD47 antibodies that directly induce tumor cell death are being explored as potential cancer therapeutics [177,178].

TSP1 was also identified as a secreted protein that prevented the escape of cancer cells from the senescence induced by chemotherapy [179]. This activity of TSP1 was also CD47-dependent.

### 5.3. Tumor Initiating/Stem Cells

**Figure 4:**
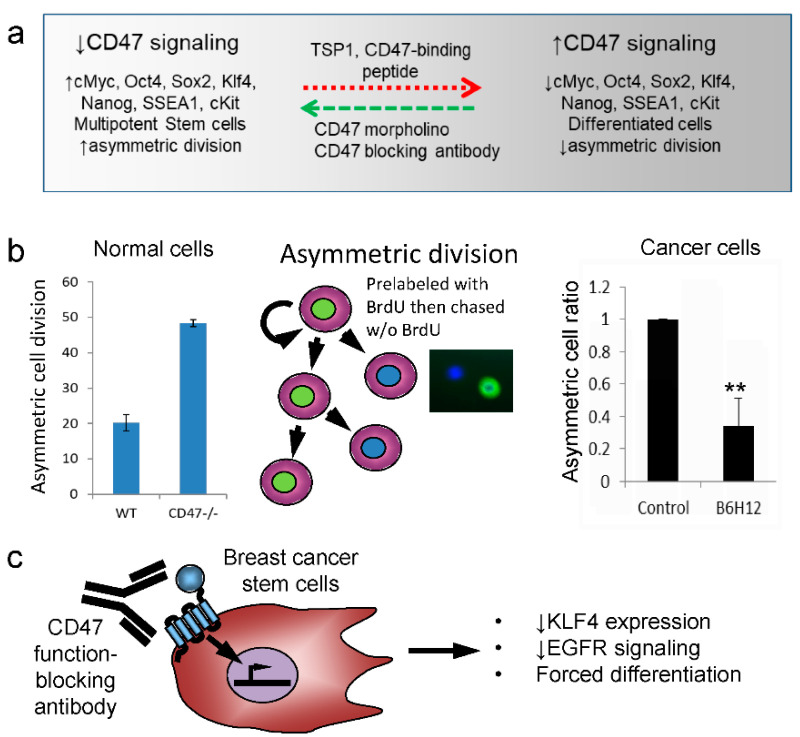
Differential effects of CD47 signaling in normal versus cancer stem/tumor-initiating cells [183]. TSP1 signaling via CD47 limits the maintenance of stem cells in nonmalignant tissues (**a**). Blocking CD47 signaling has opposing effects on asymmetric cell division in normal versus cancer cells (**b**). A function-blocking CD47 antibody forces the differentiation of breast cancer stem cells, in part by decreasing expression of the stem cell transcription factor KLF4 and decreasing epidermal growth factor receptor (EGFR) signaling (**c**).

We found that decreasing CD47 expression or TSP1-dependent CD47 signaling increases the stem cell character of non-transformed cells in vitro and in vivo [180] (Figure 4a). Thus, we were initially puzzled by reports that some cancer stem cells have elevated CD47 expression. Such high CD47 expression was proposed to protect cancer stem cells from SIRPα-dependent macrophage clearance [181], but a cell-autonomous signaling function of CD47 in cancer stem cells was not considered [147]. This prompted Lee et al. to examine CD47 in hepatocellular carcinoma, and they reported that decreasing CD47 expression using siRNA suppressed the stem cell characteristics of hepatocellular tumor-initiating cells [182].

We examined CD47 signaling in breast cancer stem cells and reached a similar conclusion [184]. Treating CD44^hi^/CD24^low^ cancer stem cells with the CD47 blocking antibody B6H12 suppressed asymmetric division (Figure 4b), stem cell transcription factors including Klf4 (Figure 4c) cell growth, and induced executioner caspase-7 activity. We used a CRISPR strategy to knock out CD47 in these cancer stem cells and, remarkably, found no viable CD47-null colonies could be obtained. This contrasts with using the same CRISPR/guide in non-transformed human cells, where abundant colonies of CD47-null cells were routinely obtained with the expected induction of stem cell markers. Thus, CD47 signaling limits stem cell character in non-transformed cells but is necessary to maintain stem cell characteristics in breast cancer stem cells [183]. These observations suggest that breast cancer stem cells are sustained by CD47 signaling, which could provide additional therapeutic opportunities to limit tumor growth by targeting CD47 independent of its passive “don’t eat me” function.

B6H12 treatment also acutely inhibited EGF-induced EGFR tyrosine phosphorylation in breast cancer stem cells but not in differentiated breast cancer cells [184]. Coimmunoprecipitation suggested that this is mediated by a lateral interaction between CD47 and EGFR.

Consistent with these studies, in vivo immune selection of Lewis lung carcinoma (LLC) for resistance to vaccination using tumor-primed dendritic cells resulted in increased expression of CD47 and the stem cell transcription factors Myc, Klf4, Sox2, and Oct4 [185]. Treatment of the P3 selected LLC cells with 2.2 nM TSP1 suppressed expression of the same stem cell factors, decreased their spheroid formation, and increased their sensitivity to killing by tumor-specific CTL. Pre-treatment with TSP1 similarly suppressed proliferation and spheroid formation of A549 lung carcinoma, HCT116 colorectal carcinoma, and HeLa cervical carcinoma cells. This suggests that TSP1 signaling mediated by CD47 may generally suppress cancer stem cells (CSC) and restore sensitivity to adaptive immune clearance.

## 6. Regulation of the Redox Environment, Autophagy, Metabolism

### 6.1. Role in Hypoxia Responses

Hypoxia and oxidative stress in the tumor microenvironment drive molecular signaling that supports carcinogenesis through multiple mechanisms. The molecular signaling influenced by hypoxia is mediated in part by hypoxia-inducible transcription factors (HIFs) that become elevated because oxygen tension is low in tissues or tumors [186]. The activation of HIFs controls TSP1 expression during low oxygen tension. TSP1 mRNA and protein expression are elevated in the first six hours after induction of hypoxia and can remain elevated under hypoxia for 72 h in human umbilical vein endothelial cells (HUVEC) and bovine aortic endothelial cells [187]. Increased TSP1 was reported in a murine pulmonary arterial hypertension model following exposure of the animals to chronic hypoxia [188]. Expression of the TSP1 receptor CD47 was also increased during these conditions and mediated superoxide production in an eNOS-dependent manner. Two hypoxia response elements were localized between positions −1120 and −1196 and −249 and −225 relative to the transcription starting site of *THBS1*, suggesting that HIFs directly increase transcription of *THBS1* during hypoxia. Further studies using luciferase reporter vectors of HIF1α and HIF2α found that hypoxia-mediated pulmonary TSP1 was mediated by HIF2α [188]. The implication of the increased TSP1 expression under hypoxia observed in this study was an augmented migration of fibroblasts and pulmonary artery smooth muscle cells, which disrupted the endothelial cell voltage-gated channels, leading to endothelial cell dysfunction.

The regulation of TSP1 by hypoxia seems to differ in the tumor microenvironment. Expression of TSP1 was increased in colorectal cancer cell lines and tumors lacking HIF1α and HIF2α, which was associated with reducing pro-angiogenic factors and reducing tumor angiogenesis [189]. Conversely, TSP1 was not detectable in hypoxic areas of tumors formed by mouse embryo fibroblasts transformed using E1A and H-Ras, whereas vascular endothelial growth factor expression increased in the hypoxic areas [190].

TSP1 is also upregulated during tumor invasion, which is inconsistent with the inverse relationship observed with the expression of pro-angiogenic factors in hypoxic areas. These paradoxical effects may be due to receptor expression or levels of upregulation of TSP1. These changes can also be attributed to different binding affinities of TSP1 for its receptors. At concentrations of 2 nM, TSP1 binds selectively to CD47 and stimulates superoxide production in vascular smooth muscle cells mediated by NADPH oxidase 1 [191]. Knockout studies indicated that activation of NOX1 and superoxide production are mediated by TSP1 binding to CD47 [192]. At 10-20 nM concentrations, TSP1 induced cell death of mouse cortical neurons [193]. At similar concentrations, TSP1 caused an increase in NOX and superoxide production in macrophages that were not mediated by CD47 [32]. Furthermore, nanomolar levels of TSP1 modulate NO signaling by inhibiting the fatty translocase activity of CD36, which implicates TSP1 interactions in the modulation of fatty acid metabolism and cellular energetics [194].

### 6.2. TSP1 Regulation of Metabolism

In addition to its roles in hypoxia and oxidative stress, TSP1 broadly regulates metabolism in the tumor microenvironment. In a mouse model examining the effect of diet on colorectal tumor formation in wild type and *Thbs1^−/−^* mice, we observed broad systemic changes that can modify carcinogenesis [195]. We observed changes in tricarboxylic acid (TCA) cycle metabolites citrate and isocitrate in livers of *Thbs1^−/−^*:*Apc^Min/+^* mice fed a high-fat diet compared to *Apc^Min/+^* mice fed the same diet. A comparison of WT and *Thbs1^−/−^* mice also showed increases in α-ketoglutarate, succinate, fumarate, and malate, implicating TSP1 as a negative regulator of TCA metabolites. In this study, feeding with a high-fat diet caused the expected increases in free fatty acids among groups, but specific changes in the *Thbs1^−/−^*:*Apc^Min/+^* group included increases in several medium-chain free fatty acids and lack of growth in some long-chain fatty acids such as oleate. *Thbs1^−/−^*:*Apc^Min/+^* mice fed a low-fat diet exhibited an ~2-fold increase in the ketone body 3-hydroxybutyrate relative to the low fat diet-fed *Apc^Min/+^* mice. 3-hydroxybutyrate is a serum biomarker of colorectal carcinoma and other cancers and may explain the increase in tumor multiplicity observed in this model driven by the loss of TSP1 [195].

Several studies showed that the expression of TSP1 is regulated by changes in metabolism, including upregulation mediated in part by high levels of glucose in diabetic models and correlated with hyperglycemia in human patients [196,197,198]. Consistent with this, a reduction in lactate levels in glioma cells was associated with decreased TSP1 levels and subsequent reduction in the migration of these cells [199]. Conversely, the addition of lactate increased TSP1 levels and promoted glioma cell migration by the TSP1-mediated regulation of TGF-β2. The metabolic regulation of TSP1, as explained above, is context-dependent. Whether TSP1 alters metabolism to promote or inhibit carcinogenesis will depend on the cancer type and which TSP1 receptors are expressed and activated.

Effects of TSP1 on cell metabolism in the tumor microenvironment can be extrapolated from studies examining the role of CD47 on cellular bioenergetics. CD47 deficiency has radioprotective effects on T cells by modulating metabolism [200]. Radiation-induced metabolic changes associated with CD47 blockade include increased glucose uptake and glycolysis, increased nucleotide biosynthesis levels, and preserved TCA cycle metabolites. Loss of CD47 enhanced mitochondrial function, increased methyl group donors (creatinine, 5-methyl tetrahydrofolate, and choline) and modulated antioxidant responses to glutathione levels compared to WT cells. Similar effects were seen in irradiated mouse lungs of WT and *Cd47^−/−^* animals, with increased glutathione levels, regulation of lipid metabolism with a decrease in fatty acid metabolites, increased phospholipid production, and preservation of nucleotide biosynthesis [201].

These effects suggested that TSP1 or CD47 blockade could cause resistance to cancer treatment using cytotoxic agents. Lack of expression of TSP1 is associated with resistance to taxane-based chemotherapy in lung adenocarcinoma, whereas siRNA depletion of CD47 resulted in cells’ sensitization to taxanes [202]. In a murine model of breast cancer resistance to tamoxifen, targeting the unfolded protein response by ablation of the chaperone GRP78 restored tamoxifen sensitivity [203]. This sensitivity was associated with the regulation of fatty acid oxidation and a reduction in CD47 expression. The addition of linoleic acid restored sensitivity to tamoxifen and reduced CD47 levels associated with increased macrophage clearance of tumors. In subsequent studies, evaluation of tumors by TSP1 immunostaining indicated that its expression was increased in the tumors re-sensitized to tamoxifen [204], thus indicating an interplay of TSP1, CD47 expression, and metabolic regulation on cancer therapy sensitivity. The sensitivity to death by cancer therapy seems paradoxical given the protective roles of TSP1 or CD47 deletion on tissue survival of injuries caused by ischemia or ionizing radiation [37].

### 6.3. Regulation of Cell Stress Responses and Autophagy

CD47 plays a role in cell fate during times of cellular stress, including promoting the survival of cells exposed to ionizing radiation. Cell viability and proliferative capacity of a CD47-deficient Jurkat T cell mutant were preserved following irradiation compared to wild-type cells [205]. Improved cell survival was associated with increased autophagy. Autophagy is a highly conserved catabolic process that maintains cellular homeostasis by facilitating lysosomal degradation of intracellular protein and organelles. Increased autophagosome formation was observed by electron microscopy and by high LC3 fluorescence. Furthermore, protein expression of LC3-B was increased, with a decrease in p62, indicating that blockade of CD47 increased autophagic flux. Thus, increased survivability is associated with activation of autophagy. Expression of autophagy-related proteins ATG5, ATG7, and Beclin-1 was also increased in CD47-deficient Jurkat cells and in vivo in lungs from irradiated mice treated with a CD47 antisense morpholino. The protective effects of reducing or eliminating CD47 expression were reversed by treatment in combination with 3-methyladenine and hydroxychloroquine or following knockdown of ATG5/7 expression.

Further studies showed that the blockade of CD47 protected cardiac tissue and cells from the death and loss of function associated with anthracycline treatment [206]. The protective mechanism was mediated by activation of autophagy. Remarkably, systemic blockade of CD47 in mice resulted in the sensitization of tumors to anthracycline therapy, which was associated with increases in calreticulin and high molecular group box 1 (HMGB1). This indicates the activation of immunogenic cell death as a potential mechanism of sensitization to chemotherapy. Presumably, TSP1 treatment would reverse the regulation of autophagic flux through CD47; however, whether TSP1 would enhance or inhibit autophagy is not entirely understood. TSP1 activated autophagy in H-Ras expressing cancer cells [207]. The regulation by TSP1 was deduced by using the CD47-binding TSP1-derived peptide 4N1K at high concentrations, which may not reflect the physiological activities of TSP1. Still, increased LC3 protein expression was associated with a reduction in tumor growth. Correspondingly, other studies showed that TSP1 treatment increases age-related blood-brain barrier leakiness by activating the p62/sequestosome-1 binding of tight junction proteins, mediating their autophagosomal degradation [208]. Although these studies implicate TSP1 in the regulation of autophagy, defining which of its receptors mediate autophagy requires further study in the context of carcinogenesis.

### 6.4. Regulation of Metabolism and Mitochondrial Stress in T and NK Cells

The ability of TSP1 signaling via CD47 to inhibit Myc levels in T cells [180] may contribute to metabolic reprogramming that limits cytotoxic T cell function in the tumor microenvironment. Activated T cell proliferation and cytokine production depend on enhanced glucose metabolism [209,210]. The transient glucose restriction in activated CD8^+^ T effector cells metabolically primes effector functions and enhances tumor clearance in mice [210]. The transcriptional activity of Myc and HIF-1 are both upregulated in response to T cell activation and lead to upregulation of genes encoding enzymes that promote glycolysis, such as pyruvate kinase (*PKM1*), hexokinase 2 (*HK2*), and *GLUT1* [209,211,212,213,214]. These changes promote metabolic reprogramming in tumor-infiltrating immune cells [215,216].

We found that CD47 expression in the tumor microenvironment also regulates NK cell recruitment and transcriptional responses to activating stimuli, and its absence results in a defect in mitochondrial metabolism [164]. Global gene-expression analysis revealed that metabolism and ion transport were upregulated in association with a higher basal glycolytic flux assessed by extracellular acidification rate values but not the basal mitochondrial flux assessed by the oxygen consumption rate in *Cd47^−/−^* NK cells. Thus, higher metabolic activity and mitochondrial stress responses of CD47-deficient NK cells result in more intracellular ROS, which could account for the observed NK cell exhaustion profile identified by gene expression profiling [164]. This mechanism may account for the depletion of NK cells in lymphocytic choriomeningitis virus (LCMV)-infected *Cd47^−/−^* mice and in tumor-bearing *Cd47^−/−^* mice [163,164].

### 6.5. DNA Damage Responses

Cells lacking CD47 exhibited more rapid repair of dsDNA strand breaks induced by ionizing radiation [217]. Faster repair of DNA damage is probably supported by the increased induction of anabolic pathways that generate the nucleotides we observed in CD47-deficient cells after exposure to ionizing radiation [200]. Furthermore, CD47 expression selectively sensitized Jurkat T cells to specific inhibitors of topoisomerases, which are known targets of schlafen-11 (SLFN11), and to class I histone deacetylase (HDAC) inhibitors [217]. SLFN11 expression in human cancers is positively correlated with sensitivity to genotoxic agents, including topoisomerase inhibitors [218,219,220,221,222,223,224]. Loss of *SLFN11* expression in cancer cells involved hypermethylation of its promoter and epigenetic changes in histone acetylation [225,226]. Correspondingly, expression of *SLFN11* in resistant cancer cell lines induced by class I HDAC inhibitors restored their sensitivity [225].

CD47 mRNA expression is positively correlated with SLFN11 mRNA expression in a subset of human cancers but not in the corresponding nonmalignant tissues in TCGA [217]. CD47 knockdown, gene disruption, or treatment with a CD47 function-blocking antibody decreased SLFN11 expression in Jurkat cells. TSP1 also suppressed SLFN11 expression in WT but not CD47-deficient T cells [217]. Re-expressing SLFN11 restored radiosensitivity in CD47-deficient cells. Disruption of CD47 in PC3 prostate cancer cells similarly decreased SLFN11 expression and was associated with a CD47-dependent decrease in acetylation and increased methylation of histone H3 in the SLFN11 promoter region [217]. CD47 mRNA expression was also negatively correlated with SLFN11 promoter methylation in some tumors [217]. The ability of HDAC or topoisomerase inhibitors to induce SLFN11 expression in PC3 cells was lost when CD47 was targeted in these cells. Disrupting CD47 in PC3 cells also increased resistance to etoposide. These data identify CD47 as a context-dependent regulator of SLFN11 expression and suggest an approach to improving radiotherapy and chemotherapy responses by combining these treatments with CD47-targeted therapeutics.

### 6.6. Clearance of Dead/Dying Cells

Early studies by Savill and colleagues identified TSP1 as a bridging molecule that targets apoptotic cells for clearance by macrophages, involving TSP1 interactions with αvβ3 integrin and CD36 on phagocytes [36]. TSP1-dependent clearance of apoptotic cells was subsequently implicated in an anti-inflammatory activity that protected mice from endotoxic shock [227]. Recent studies extended this pro-phagocytic activity of TSP1 to the clearance of aged red blood cells and implicate clustered CD47 as the conformation-dependent TSP1 receptor on aged red blood cells [52,228]. Correspondingly, TSP1 enhanced the phagocytic activity of macrophages in vitro, and loss of *Thbs1* decreased the phagocytic activity targeting muscle cells of male dysferlinopathic BlaJ mice [229] and in a fixed hindlimb muscle ischemia model [230]. The relevance of these studies to the tumor microenvironment remains to be defined, but the recently reported TSP1-enhanced phagocytosis of hepatocellular carcinoma cells is consistent with this mechanism [231].

## 7. Regulation of Intercellular Signaling Mediated by Extracellular Vesicles

**Figure 5:**
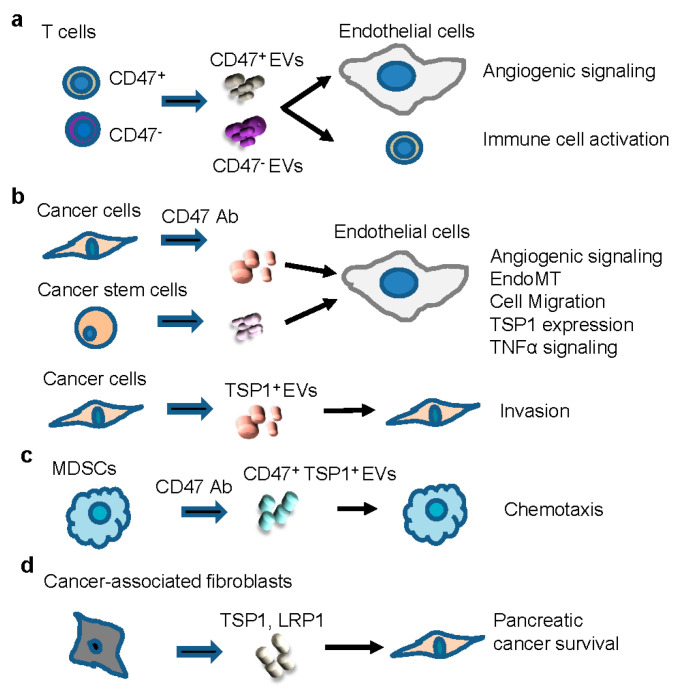
Roles of TSP1 and CD47 in the intercellular communication mediated by EVs in the tumor microenvironment. EVs derived from T cells (**a**) modulate angiogenic signaling in endothelial cells and activate other T cells in a CD47-dependent manner. EVs produced by cancer cells and cancer stem cells (**b**) have TSP1- and CD47-dependent effects on endothelial cells and other tumor cells that CD47 antibodies can modulate. EVs enriched with TSP1 from myeloid-derived suppressor cells (MDSCs, (**c**)) stimulate chemotaxis, blocked by a CD47 antibody. EVs produced by cancer-associated fibroblasts (**d**) containing TSP1 associated with LRP1 are circulating poor survival markers.

EVs released by malignant cells mediate multiple types of intercellular communication in the tumor microenvironment (Figure 5) and can disseminate via the circulation to initiate the formation of a metastatic niche [232,233]. Tumor EVs function by transporting macromolecules, including mRNAs, miRNAs, other non-coding RNAs, and proteins with oncogenic roles in the tumor microenvironment [234]. We identified a new role for CD47 in regulating intercellular communication mediated by EVs released by T cells [109]. The mRNA content in EVs differed between WT and CD47-deficient cell lines, and uptake of those EVs by target cells led to CD47-dependent changes in gene expression in target cells that regulate angiogenesis and immune cell function (Figure 5a). These initial findings led us to investigate further the role of CD47 in EVs produced by normal and malignant cells and how CD47 regulates which RNAs are packaged into EVs.

### 7.1. Regulation of Vascular Cells

Treatment of HUVEC with EVs from MDA-MB-231 breast carcinoma cells inhibited the VEGF signaling pathway [184]. EVs derived from triple-negative versus ER^+^/PR^+^ breast carcinoma cells differentially altered expression of VEGFR2, AKT3, AKT2, and HIF in HUVEC corresponding with their tumorigenic potential (Figure 5b). This correlated with a higher abundance of CSCs in MDA-MB-231 relative to T47D1 cells. CD47^high^ EVs derived from CSCs were taken up more by HUVEC than were CD47^low^ EVs. Notably, their uptake was not attenuated by a CD47 blocking antibody. Treatment of HUVEC with breast CSC-derived EVs decreased TSP1 mRNA in HUVEC, and a CD47 function-blocking antibody reversed that inhibition [235].

Another study of MDA-MB-231 EVs identified a role for TSP1 associated with EVs in promoting the invasion of the cancer cells through a HUVEC monolayer [236]. Analysis of TSP1^low^ and TSP1^high^ EVs derived from knockdown and re-expression approaches from MDA-MB-231, and MCF7 cells revealed that TSP1 promotes cell migration in MCF7 cells and reduced in MDA-MB-231 cells in vitro, as well as in a zebrafish model. EV-derived TSP1 impaired the integrity of the endothelial layer by lowering the expression of the intercellular junction molecules ZO-1 and VE-cadherin. Some of the EV-induced changes in HUVEC mRNA expression were reversed by the peptide LSKL, inhibiting activation of latent TGFβ by TSP1. This suggested that TGFβ signaling mediates some effects of EV TSP1 to disrupt endothelial intercellular junctions.

Additional studies have identified TSP1 protein in EVs released by malignant cells. EVs derived from nasopharyngeal (NP) carcinoma cells enhanced angiogenic signaling by increasing tubulogenesis, migration, and invasion of HUVEC in a dose-dependent manner as compared to EVs from immortalized nasopharyngeal epithelial cell lines [237]. Proteomic analysis of these EVs identified upregulation of some proangiogenic proteins and reduced TSP1 in NP carcinoma EVs compared to nasopharyngeal epithelial cell EVs. This suggested that angiogenic signaling mediated by tumorigenic EVs may be due to loss of the suppressive effect of TSP1. Similarly, TSP1 in EVs was reduced after MDCK kidney epithelial cells were transformed by oncogenic H-Ras [238]. Conversely, treatment of non-small cell lung carcinoma cells with pigment epithelium-derived factor increased TSP1 levels in released EVs, which inhibited the motility and invasion of the cells [239].

### 7.2. Regulation of Immune Cells

TSP1 associated with EVs released by cancer cells may also modulate antitumor immunity. TSP1 and CD47 were identified on the surface of EVs released by myeloid-derived suppressor cells (MDSCs) induced in mice by implanted 4T1 mouse mammary carcinoma [129]. The EVs stimulated chemotaxis of MDSCs, which was inhibited by CD47 and TSP1 function-blocking antibodies (Figure 5c). The CD47 counter-receptor SIRPα was identified on MDSCs but not on the released EVs, whereas TSP1 was enriched 12-fold on EVs compared to the parental MDSCs.

EVs released by wild-type versus CD47-deficient T cells altered the sensitivity of recipient T cells to the inhibition of TCR-mediated activation by TSP1 in a CD47-dependent manner [109]. Activation of T cells with anti-CD3 strongly induced TSP1 mRNA and increased TSP1 expression on the cell surface in wild-type cells but not in a CD47-deficient T cell mutant. However, the expression of TSP1 on the released EVs did not require CD47.

### 7.3. Cancer-Associated Fibroblasts

TSP1 is associated with EVs released by cancer-associated fibroblasts via a complex that includes LRP1 and annexin A6 [41] (Figure 5d). Depletion of these EVs decreased metastasis in a pancreatic cancer model, and high circulating levels of these EVs were associated with decreased survival in pancreatic cancer patients.

## 8. TSP1 and Carcinogenesis

Several studies support roles for TSP1 in carcinogenesis. Targeted overexpression of TSP1 in the epidermis delayed and reduced premalignant epithelial hyperplasias induced by chemical carcinogens [240]. This protective role of TSP1 over-expression was extended to UVB-induced skin carcinogenesis [130] and spontaneous mammary adenocarcinomas in TgN-neu mice [241]. Conversely, loss of *Thbs1* increased mammary adenocarcinomas in these mice [241] and increased osteosarcoma incidence but not the incidence of some other malignancies in mice lacking p53 [19]. In a murine model of prostate cancer metastasis to bone, increased TSP1 was observed in platelets, and implantation of tumors in *Thbs1* null mice resulted in increased tumor size [242]. On the other hand, the absence of TSP1 reduced bone marrow-derived cell mobilization and enhanced osteoclast formation, resulting in decreased tumor-induced bone formation, suggesting a role of TSP1 in the pre-metastatic niche formation.

TSP1 limited angiogenesis and inflammatory responses that contribute to colorectal carcinogenesis in *Apc^Min/+^* mice [243] and colorectal carcinogenesis induced by chronic inflammation [244]. The ability of TSP1 to regulate the responses of cells and tissues to stress prompted us to examine whether loss of TSP1 also has systemic effects on metabolism that modulate carcinogenesis [195]. *Apc^Min/+^:Thbs1^−/−^* mice exhibited decreased survival and higher tumor multiplicities in the small and large intestine relative to *Apc^Min/+^* mice when fed a low-fat Western diet. However, the protective effect of endogenous TSP1 was lost when the mice were fed a high-fat Western diet. Biochemical profiles of liver tissue identified systemic metabolic changes associated with the effects of TSP1 and dietary lipid intake on tumorigenesis. A high-fat Western diet differentially regulated amino acid, energy, and lipid metabolism in *Apc^Min/+^:Thbs1^−/−^* mice relative to *Apc^Min/+^* mice. Changes in ketone body and tricarboxylic acid cycle intermediates identified functional interactions between Apc and TSP1 signaling that control mitochondrial function. These data suggest that the protective role of TSP1 to limit adenoma formation in *Apc^Min/+^* mice results in part from improved mitochondrial function and eicosanoid signaling [245].

## 9. Development of TSP1 Derived Agents for Anti-Tumor Therapy

The characterization of TSP1 as a potent inhibitor of angiogenesis led to efforts to harness this activity for anti-tumor therapies by targeting CD36 [43,44,45,46,47]. However, as discussed above and reviewed elsewhere, the multifaceted role of TSP1 in the tumor microenvironment may compromise the intended therapeutic effect [246,247]. Three recently reported approaches differ mechanistically from past abandoned clinical approaches, some of which focus on beneficial modulation of TSP1-CD47 interactions.

Consistent with the anti-angiogenetic and antitumor activities of TSP1-mimetic peptides ABT-510 and ABT-898 [248], one approach increased the concentration of anti-angiogenic fragments of TSP1 in the tumor microenvironment. Tumor regression and vascular normalization were observed in a syngeneic orthotopic mouse model of advanced-stage epithelial ovarian cancer treated daily with recombinant type 1 repeats (3TSR), including or without fusion to a CD47-binding sequence (FYVVMWK, 4N1), and expressed from adeno-associated virus (AAV) vectors [249]. A single administration of the AAV agents resulted in durable expression of the TSP1-derived products for at least 30 days. AAVs expressing 3TSR alone or with the CD47-binding sequence resulted in marked tumor reduction. However, only the 3TSR AAV enhanced survival at 60 days. The 3TSR AAV was associated with a greater survival benefit and prevention of second lesions than the CD47-binding peptide AAV, despite the combination AAV being the most active in vitro. The authors speculate that the differential in vivo efficacy of the 3TSR vs. CD47-binding peptide could be due to the loss of vector transduced cells in the latter resulting in a suboptimal level of expression. However, 4N1 has questionable CD47 functional specificity based on its demonstrated activity on CD47 deficient cells [107]. These studies support the continued exploration of TSP1-derived agents as second-generation antiangiogenic or vascular-normalizing therapies. This could be especially relevant in combination with immunotherapies benefiting from improved tumor-immune cell infiltration.

CD36 has reemerged in anti-tumor therapy as an immunotherapy target on macrophages and T cells [250]. CD36 is selectively expressed on intratumoral regulatory T cells and enables them to metabolically adapt to the lactic-acid enriched tumor microenvironment by increasing fatty acid uptake. Combining anti-PD-1 therapy with an anti-CD36 antibody in a syngeneic mouse melanoma model effectively reduced tumor burden than either agent alone. TSP1 and the parent peptide upon which the Abbott CD36 drug ABT-510 was based inhibit fatty acid uptake. Although ABT-510 does not significantly inhibit fatty acid uptake [251], future TSP1-derived therapeutic agents that potently inhibit CD36 fatty acid transport could enhance anti-tumor immunity and improve T cell-targeted combination therapies.

A recent effort to therapeutically inhibit TSP1 interaction with CD47 utilized in silico tools to identify a putative TSP1 interaction surface on CD47. This surface was used to design a cyclic peptide (TAX2) that reportedly binds to TSP1 with low micromolar affinity [252]. Principally characterized for antiangiogenic activity in vitro, TAX2 activity was sensitive to CD36 blockade. The authors proposed that the activity of TAX2 is mediated by liberating TSP1 from its high-affinity interaction with CD47 and promoting its interaction with CD36. TAX2 reduced tumor burden in a mouse syngeneic melanoma model, reducing tumor vascularization. Consistent with its TSP1-CD47 inhibitor activity, TAX2 also exhibited antithrombotic activity [253]. While these studies suggest the potential value of TSP1-CD47 disrupting agents, the TAX2 peptide falls short of demonstrating the specificity and potency needed as a robust research tool and bone fide pretherapeutic agent.

## 10. Conclusions

The apparent contradictions that have emerged from efforts to define the role of TSP1 in the tumor microenvironment are understandable considering its interactions with multiple signaling receptors and with angiogenic and immune-modulatory factors in the extracellular matrix. To date, efforts to develop therapeutics have targeted the TSP1 receptors CD36 and CD47. Although TSP1 mimetics targeting CD36 showed antitumor efficacy in mice and dogs, these have not proven effective in human clinical trials [45,46,47]. CD47-targeted antibodies have shown more promising results in human clinical trials [54,148,149,150,151], but it remains unclear whether modulating TSP1 signaling plays any role in their efficacy. However, preclinical studies using the CD47 antibody B6H12 indicate that some CD47 antibodies can block both TSP1 and SIRPα interactions with CD47 [54]. Next-generation CD47-targeted agents that selectively disrupt the TSP1-CD47 versus the CD47-SIRPα interaction are needed to differentiate the roles of each in CD47 receptor biology and remain a focus of our ongoing work [254].

A persistent challenge to developing therapeutics targeting the TSP1-CD47 interaction is the lack of molecular details for this interaction. Structural data, perhaps enabled by increasingly high-resolution cryo-EM techniques, could define the critical interaction interface, which would help develop therapeutic antibodies, nanobodies, and small molecule antagonists that selectively block TSP1 interactions with CD47.

Another strategy that we are pursuing is to knock down CD47 expression in the tumor microenvironment using antisense morpholino oligonucleotides, which limit both TSP1- and SIRPα-CD47 signaling. These morpholinos have proven effective in limiting tumor growth in immune-competent hosts when combined with radiotherapy, chemotherapy, or an immune checkpoint inhibitor targeting CTLA4 on T cells [113,114,206].

Continued investigation is also needed to define which interactions of TSP1 in the tumor microenvironment play dominant roles in regulating tumor growth, metastasis, and sensitivity to host immune surveillance. This knowledge could guide the development of receptor-specific targeting strategies. Future therapies will undoubtedly need to combine elements of TSP1-signaling agonists with antagonists to effectively balance its pleiotropic effects on tumor growth and anti-tumor immunity. Based on the divergent clinical correlations between TSP1 expression and prognosis in different cancer types, optimal TSP1-directed therapies may be cancer type-specific.

## Figures and Tables

**Table 1:** Cell surface TSP1 receptors expressed on cancer and stromal cells.

Integrin	Function	Reference
α3β1	Pro-angiogenic, cancer cell adhesion/motility	[26,27,28,31]
α4β1	Pro-angiogenic, T cell chemotaxis and MMP expression	[23,25]
α6β1	Pro-angiogenic, macrophage ROS	[24,32]
α9β1	Pro-angiogenic	[29]
αvβ1	Vascular remodeling	[33]
αvβ3	Tumor cell adhesion	[34]
CD36	Anti-angiogenic; phagocytosis of apoptotic cells	[35,36]
CD47	Anti-angiogenic, immune checkpoint, stress responses	[10,37]
CD148	Anti-angiogenic, EGFR regulation	[38,39]
Calreticulin/LRP1	Adaptive immunity, cancer-associated fibroblasts, EVs, metastasis	[40,41]
STIM1	Calcium entry	[42]
